# Response of soil microbial compositional and functional heterogeneity to grazing exclusion in alpine shrub and meadows in the Qinghai–Tibet Plateau

**DOI:** 10.3389/fmicb.2022.1038805

**Published:** 2022-11-30

**Authors:** Shilin Wang, Theophilus Atio Abalori, Wenhu Wang, Xiuxia Deng, Wanting Liu, Jinlan Wang, Wenxia Cao

**Affiliations:** ^1^College of Pratacultural Science, Gansu Agricultural University, Lanzhou, China; ^2^Key Laboratory of Grassland Ecosystem, Ministry of Education, Lanzhou, China; ^3^Qinghai Academy of Animal Science and Veterinary Medicine, Qinghai University, Xining, China

**Keywords:** soil microbes, metagenomics, heterogeneity, microbial diversity, woody meadow, grazing management

## Abstract

Soil microorganisms found in shrub-meadow ecosystems are highly heterogeneous and extremely sensitive to grazing, but changes in microbial compositional and functional heterogeneity during grazing exclusion (GE) have been largely overlooked compared to community diversity. We collected soil samples from heavily grazed plots (6.0 sheep/ha) and GE plots (matrix and patch areas in both), and used a combination of next-generation sequencing, vegetation features, and the associated soil property data to investigate the effect of GE on the composition and function of microbial communities (bacteria fungi, and archaea) in 0–10 cm soils. Regarding community composition, the proportions of species in bacteria, fungi, and archaea were 97.3, 2.3, and 0.4%, respectively. GE significantly affected the species diversity of fungi and archaea but not that of bacteria. GE decreased the heterogeneity of bacteria (2.9% in matrix and 6.2% in patch) and archaea (31.1% in matrix and 19.7% in patch) but increased that of fungi by 1.4% in patch. Regarding community function, enzyme diversity and heterogeneity were increased by 10.4 and 9.4%, respectively, in patch after 6 years of fencing, exemplifying a high level of microbial functional redundancy. The Kyoto Encyclopedia of Genes and Genome pathways—cell growth and death, translation, digestive system, and nucleotide metabolism—were functional biomarkers (linear discriminant analysis effect size method) in matrix-non-grazed plots, whereas lipid metabolism, xenobiotics biodegradation and metabolism, and metabolism of terpenoids and polyketides, cell motility, cancer: overview, endocrine system, and membrane transport were biomarkers in patch-non-grazed plots. Additionally, GE improved the capacity for fatty acid metabolism but decreased the abundance of methane-producing archaea by 42.9%. Redundancy analysis revealed that the factors that affected microbial composition the most were soil aggregates, soil moisture, and the number of plant species, whereas those that affected microbial function the most were soil available phosphorus, soil temperature, and shrub canopy diameter. Our results quantified soil microbial heterogeneity, emphasizing the different responses of the composition and function of bacteria, fungi, and archaea to GE in alpine shrubs and meadows.

## Introduction

Soil microbes play decisive roles in the regulation of vegetation recovery by shaping the plant–soil feedback (Hannula et al., 2021; Inderjit et al., 2021). Through soil feedback, microbes potentially contribute to plant community assembly through spatial self-organization (Ren et al., 2021; Yurek et al., 2021). Microbial communities are spatially heterogeneous in all natural ecosystems, which is a result of adaptation to local environmental conditions and stochastic processes (Uritskiy et al., 2020). Experiments on both the microcosm and global observational scales suggest that microbial diversity is associated with the functional diversity of ecosystems (Maherali and Klironomos, 2007; Delgado-Baquerizo et al., 2016; Semchenko et al., 2018), implying that microbial communities with greater richness perform better. However, heterogeneity is an important feature of biodiversity because greater divergence increases the likelihood of some species performing functions under conditions of temporal and spatial variation and buffering functions against the loss of taxa, thereby maintaining ecosystem function (Loreau, 2004).

Soil microbial heterogeneity is shaped by both plant communities and soil properties (Shi et al., 2020; Upton et al., 2020). Plant diversity is possibly the primary regulator of soil microbial processes in response to environmental changes (Steinauer et al., 2015). Simultaneously, environmental heterogeneity is a mechanism that determines microbial community composition. Fencing usually leads to increased nutrients in soil, which is largely due to the accumulation of plant litter, whereas soil bulk density is decreased because of removing of animal trampling (Wang et al., 2019). After long-term grazing exclusion (GE), the diversity within the bacterial, archaeal, and fungal communities was increased, and the relative abundance of Chitinophagaceae related to carbon cycle increased with the increase of soil nutrient availability in natural grassland (Wang et al., 2019). Microbes decompose soil organic matter and plant litter in reverse, regulating carbon and nitrogen cycling in soil (Schimel and Schaeffer, 2012; Aislabie et al., 2013). Grazing intensity modulates the abundance and spatial heterogeneity of microbial communities in ecological networks, with direct effects on microbial taxa heterogeneity (Eldridge et al., 2020). Soil microbes are extremely sensitive to temperature and moisture alterations in high-altitude ecosystems, which reduce functional gene richness but increase community heterogeneity (Yuan et al., 2018). Spatial heterogeneity driven by environmental conditions guarantees substantial redundancies in soil microbial functions (Shi et al., 2021). On small spatial scales, microbial distribution patterns are a legacy of historical events, such as geological evolutionary processes (Zhang et al., 2018). Microbial studies of grazing systems, compared with agricultural systems, are relatively limited, particularly in woody rangeland ecosystems (Kooch et al., 2020). The mechanisms underlying the effects of woody and herbaceous species on soil microorganisms differ widely because woody patches are more resilient owing to spatial self-organization (Rietkerk et al., 2021; Amir et al., 2022). Shrubs can shelter seedlings and animals from the harsh environment and enrich soil seedbanks (Moreno-de las Heras et al., 2016), act as fences for livestock, increase the humidity and temperature in the soil of local areas, and improve the soil moisture and nutrient content (Mihoc et al., 2016; Gao et al., 2019). However, differences in the diversity and heterogeneity of the microbial communities beneath (i.e., patch) and outside (i.e., matrix) shrub canopies remain unclear.

**FIGURE 1:**
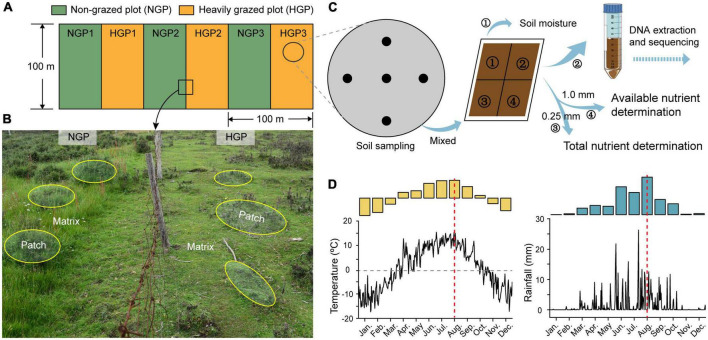
Experimental set-up. **(A)** HGPs and NGPs were set along a shady slope direction in shrub meadows in May 2014; the area was 100 m × 50 m per plot. **(B)** In this set-up, the *Potentilla fruticosa* canopy area and the shrub intercanopy spaces are considered the patch (yellow ellipses) and matrix, respectively. Four locations were designated for analysis (M-HGP, P-HGP, M-NGP, and P-NGP). **(C)** Collection of soil samples. **(D)** Temperature and rainfall measurements of the experimental area in 2020. Microenvironment and soil data were collected in August 2020 (red dotted line in **D**). See text for details.

Shrub-meadow systems, which are common in the Qinghai–Tibet Plateau, have great potential as income sources for herders. Over the past few decades, the excessive pursuit of economic benefits has caused severe degradation of shrub-meadow systems; thus, ecological restoration is particularly urgent. GE by exploiting the self-organizing capacity of shrub-meadow systems to self-regulate is an effective and natural strategy for ecological restoration. In this study, a GE area was established by fencing in a shrub-meadow system with *Potentilla fruticosa* as the dominant shrub species (Figure 1) to explore changes in the structures and functions of the soil microbial (bacterial, fungal, and archaeal) community in the matrix and patch of heavily grazed plots (M-HGPs and P-HGPs, respectively) and non-grazed plots (M-NGPs and P-NGPs, respectively). Based on the resource aggregation effects of shrubs and consequences of GE in the Qinghai–Tibet Plateau, we tested the following three hypotheses: (1) GE changes soil microbial composition and increases the compositional heterogeneity within bacteria, fungi, and archaea in patch compared to matrix. (2) GE alters soil microbial function and increases the number of enzymes and functional biomarkers in patch. (3) GE affects soil microbial communities with the recovering of shrub-herbaceous plants and their environmental characteristics, and shrub canopy diameter may be a major factor in microbial composition and function in shrub meadows.

## Materials and methods

### Site description and experimental design

The study site is located in the eastern Qilian Mountains of Zhuaxixiulong Town, Tianzhu Tibetan Autonomous County City, Gansu Province, China (37°10′11.95″ N, 102°47′10.75″ E; 3043.2 m above sea level). The climate of the study site is typical of the Qinghai–Tibet Plateau, characterized by a marked difference between daytime and nighttime temperatures. The long hours of sunlight exposure produce strong solar radiation with no absolute frost-free period throughout the year. The winter months are cold and dry. June, July, and August are the warmest months. The annual average temperature is 1.1°C. January is the coldest month, with an average temperature of –10.3°C, whereas July is the warmest month, with an average temperature of 13.7°C. The average annual rainfall is 467.9 mm, with 75.6% of rainfall occurring from June to September. The meteorological data from 2010 to 2020 were recorded at the Wushaoling National Meteorological Station in the study area. The annual plant growth period is approximately 120 days (Wang et al., 2021). The major vegetation types are alpine meadows, alpine shrublands, and alpine shrub-meadow ecotones. Deciduous and evergreen broad-leaved shrubs are the dominant shrubs, which include *Potentilla. fruticosa*, *Potentilla glabra*, *Salix rehderiana*, *Caragana jubata*, and *Rhododendron capitatum*. The dominant herbaceous plants mainly belong to the families Gramineae, Cyperaceae, and Polygonaceae, such as *Polygonum viviparum*, *Elymus dahuricus*, *Poa pratensis*, *Kobresia humilis*, and *K. capillifolia*.

A GE experimental platform was established in a shrub-meadow system in May 2014. three HGPs and three NGPs were established along a shady slope (Figure 1A). The area of each plot was 100 m × 50 m. A survey conducted before the establishment of these plots found no remarkable differences in vegetation characteristics (Supplementary Table 1). In HGPs, three Tibetan sheep, the principal local grazing animals, grazed in each plot (6.0 sheep/ha) from March to May and from September to November (rotational grazing system). The livestock was excluded throughout the year in the NGPs. In August 2020, 10 *P. fruticosa* were selected as patches, with shrub base diameter sizes of 39.5 ± 0.8 cm in each plot, and 10 points out of shrub canopies were randomly selected as matrices in each plot. All sampling points were located at a distance of >5.0 m from the fencing to avoid potential edge effects.

### Plant community and environmental data collection

A field survey of the sampling site was conducted in August 2020. 20 sampling points (10 points in patches and 10 points in matrices) were selected in each plot, 120 sampling points in total (Figure 1A). Soil temperature at depths of 0–10 cm and light intensity were measured at each point for 7 continuous days at 10:00 a.m. each day during clear weather. Vegetation and litter were surveyed and collected by quadrats (0.5 m × 0.5 m). The plant species and number were recorded. Next, 5-cm (inner diameter) soil core samples were collected from depths of 0–10 cm using an auger, and every 5 samples from the same location were mixed for a total of 24 soil samples, a quarter of which were stored at –80°C for DNA extraction and sequencing and for assessing microbiological properties. The other soil samples were air-dried and either passed through a 1.0-mm sieve screen to collect organic matter for the measurement of available nutrients or passed through a 0.25-mm sieve for the measurement of total nutrients (Bao et al., 2000; Figure 1C). In addition, 10-cm (inner diameter) soil cores were collected, packed into mesh bags, and rinsed to obtain plant roots, and 5-cm (inner diameter) soil cores were collected using a cutting ring from a depth of 10 cm and dried at 105°C to a constant weight to calculate soil moisture content and bulk density. All plant, root, and litter samples were dried to a constant weight at 70°C and weighed for calculating the biomass of above-ground plants, roots, and litter (Bao et al., 2000).

### DNA extraction and sequencing and bioinformatics analysis

Genomic DNA was extracted from soil samples of shrub-meadow systems using hexadecyltrimethyl ammonium bromide (CTAB) (Mackelprang et al., 2011; Hultman et al., 2015). Then, 1% agarose gel electrophoresis was used to detect the degree of degradation and potential contamination of DNA. The Gubit^®^ dsDNA assay kit with a Qubit^®^ 2.0 fluorometer (Life Technologies, Carlsbad, CA, USA) was used to quantify DNA concentration. The qualified DNA samples were randomly fragmented to fragment lengths of approximately 350 bp using an ultrasonicator (Covaris, Inc., Woburn, MA, USA). Next, a library was prepared by end-polishing, the addition of poly-A tails and sequencing connectors, further purification, and polymerase chain reaction (PCR) amplification. Library construction was performed using the NEBNext^®^ Ultra™ DNA Library Prep Kit for Illumina^®^ (New England Biolabs, Ipswich, MA, USA). Finally, the index-coded samples were clustered using the cBot Cluster Generation System according to the manufacturer’s instructions (Illumina, Inc., San Diego, CA, USA), the sequenced library was prepared using the NovaSeq 6000 Sequencing Platform (Illumina, Inc.), and paired-end reads were generated.

The raw data were preprocessed to improve data reliability using the Cutadapt tool^1^ to remove the connector sequences (Martin, 2011). Quality control was performed using the Trimmomatic read trimming tool (KneadData software). The effect and rationality of quality control were tested using the FastQC method (Schmieder and Edwards, 2011). After quality control, the data were aligned to the host genome using Bowtie2, and sequences not found in the host genes were preserved for subsequent analyses. Next, the species and functions were annotated based on reads. Kraken2 (the latest comparison software based on k-mer) and a self-built microbial database were used to identify the species (Wood and Salzberg, 2014; Lu et al., 2017) and Bracken was used to predict the actual relative abundance of species in the samples (Lu et al., 2017). Raw reads of all samples and databases (UniRef90) were aligned using the HUMAnN2 software. Annotation information was retrieved from relative abundance tables of multiple compositional and functional databases. The Bray–Curtis dissimilarity was used to assess differences in microbial community structures among samples, and results are presented in non-metric multidimensional scaling diagrams (Vazquez-Baeza et al., 2013). Unless otherwise noted, the parameters used in the abovementioned methods were default settings.

### Statistical analyses

The R package “vegan” was used to analyze the shared species of microorganisms among the four sampling sites. Redundancy analysis (RDA) was performed to identify potential associations between microbial communities and related factors. Based on the relative abundance of microbial species in the samples, Spearman’s correlation coefficients were analyzed and co-occurrence analysis was performed using the R package “igraph” to elucidate the correlations among species, which was conducted according to the method of Franciska et al. (2018) with slight modifications (ρ > 0.9; *p* < 0.01). In addition, functional differences among the microbial communities were analyzed [Kyoto Encyclopedia of Genes and Genome (KEGG) pathway enrichment analysis and linear discriminant analysis (LDA) effect size (LEfSe)] to identify changes in microbial function across the four sampling sites.

Heterogeneity was measured using the coefficient of variation (Eldridge et al., 2020). The data were tested for normality (Shapiro–Wilk test) and homogeneity of variances (Levene’s test). Normally distributed and homoscedastic data were analyzed using one-way analysis of variance followed by Tukey’s multiple comparison test, whereas non-normally distributed or non-homoscedastic data were analyzed using the Kruskal–Wallis test. Different lowercase letters indicate significant differences among the four sampling sites (*p*< 0.05). Because some of the environmental data (shrub canopy diameter, shrub base diameter, above-ground biomass, litter biomass, moss biomass, soil pH, light intensity, and total nitrogen) did not meet the criteria for Shapiro–Wilk test, Spearman’s correlation analysis was performed to identify correlations between plant and environmental factors (R package “corrplot”) and between factors and microbial composition and function (R package “Pheatmap”). Figures were generated using the R package “ggplot2” (version 4.1.1).

## Results

### Heterogeneous microbial community

**FIGURE 2:**
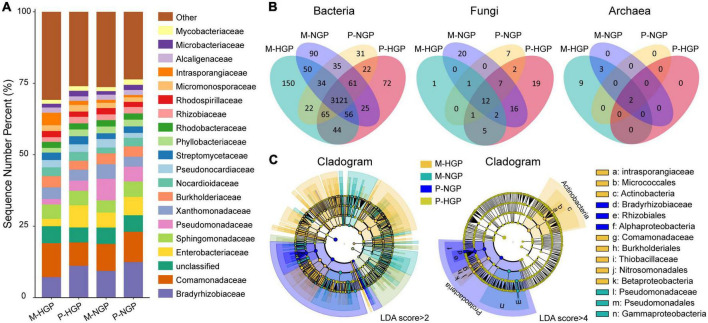
Response of microbial composition to GE. **(A)** Relative abundances of soil microbes at the family level at depths of 0–10 cm. Other classification levels are shown in Supplementary Figure 1. **(B)** Venn diagrams of shared and unique species of bacteria, fungi, and archaea. **(C)** Cladograms of microbial biomarkers at the family level identified using the LEfSe method at LDA scores of (log 10) > 2 and (log 10) > 4.

The relative abundances of resident microbial taxa were compared at the family level among the M-HGP, P-HGP, M-NGP, and P-NGP samples. The top 20 taxa accounted for 73.3% of all species, with Enterobacteriaceae, Pseudomonadaceae, Bradyrhizobiaceae, and Intrasporangiaceae being the most severely impacted by GE. The relative abundances of Bradyrhizobiaceae increased by 294.7 and 44.4% in M-NGP and P-NGP, respectively (Figure 2A). Overall, high spatial heterogeneity was observed in both HGPs and NGPs, and GE increased the relative abundance of dominant microbial populations by 6.4% in matrix and 3.2% in patch. In addition, the composition of the microbial communities was measured at different taxonomic levels. At the phylum level, Proteobacteria and Actinobacteria accounted for 96.9% of all species (Supplementary Figure 1).

Shared species analysis was performed for common bacterial, archaeal, and fungal species among the four sampling sites (M-HGP, P-HGP, M-NGP, and P-NGP), and Venn diagrams were generated (Figure 2B). The proportions of species in bacteria, fungi, and archaea were 97.3, 2.3, and 0.4%, respectively, and shared bacterial, archaeal, and fungal species among the four sampling sites accounted for 78.3, 12.9, and 14.3%, respectively. GE increased the species proportions of bacteria in the patch and matrix from 88.3 to 90.0% but decreased the species proportions of shared fungi in the patch and matrix from 30.3 to 29.4%. Archaeal species were rare, with only 14 species being detected, and they were distributed among the families Nitrososphaeraceae (*Nitrososphaera viennensis* and *Candidatus Nitrosocosmicus franklandus*), Methanobacteriaceae (*Methanobrevibacter millerae*, *Methanobrevibacter ruminantium*, *Methanobrevibacter* sp. YE315, and *Methanosphaera* sp. BMS), and Methanosarcinaceae (*Methanosarcina barkeri*, *Methanosarcina flavescens*, *Methanosarcina horonobensis*, *Methanosarcina lacustris*, *Methanosarcina mazei*, *Methanosarcina siciliae*, *Methanosarcina* sp. MTP4, and *Methanosarcina thermophila*). GE increased the proportion of shared archaeal species in the patch and matrix from 14.3 to 40%. In patches, shared bacterial, archaeal, and fungal species in the HGPs and NGPs accounted for 91.1, 30.6, and 100% of the total species, respectively. In matrices, the proportions of shared bacterial, archaeal, and fungal species decreased to 89.3, 23.1, and 35.7%, respectively.

Next, biomarkers were compared among the plots (Figure 2C). LEfSe is an algorithm used for identifying high-dimensional biomarkers from multiple taxa. For an LDA score (log 10) of >2, 323, 119, 115, and 61 biomarkers specific to the M-HGP, P-HGP, M-NGP, and P-NGP, respectively, were identified, whereas for an LDA score (log 10) of >4, the number of biomarkers decreased to 9, 0, 3, and 4, respectively. Biomarkers of the HGPs mainly comprised Actinobacteria, whereas all biomarkers of the NGPs were Proteobacteria (Figure 2C).

**FIGURE 3:**
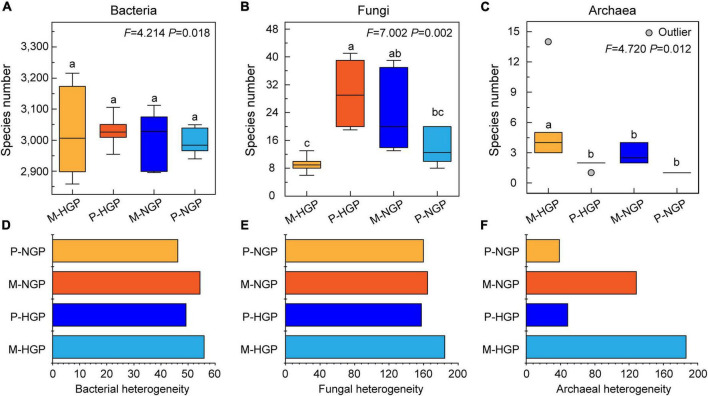
Relative abundance **(A–C)** and heterogeneity **(D–F)** of soil bacteria, fungi, and archaea. In each plot, different capital letters indicate significant differences (*p* < 0.05) in M-HGP, P-HGP, M-NGP, and P-NGP in **(A–C)**. The four sampling sites are indicated by different colors in **(A–F)**.

The analysis of soil microbial species revealed no significant differences in bacterial diversity between patch and matrix plots and no significant differences in response to GE (Figure 3A). The number of species of archaea in matrices (5.50) was higher than that in patches (1.83), but GE eliminated this difference (Figure 3C). Unlike bacteria and archaea, the fungal diversity in P-HGPs was 3.2 times higher than that in M-HGPs. GE significantly decreased fungal diversity by 53.1% in patches but increased it by 159.8% in matrices (Figure 3B). As shown in Figures 3D–F, the heterogeneity of the fungal and archaeal community composition was greater than that of bacteria. Compared with P-HGPs, the heterogeneity of bacteria, fungi, and archaea in M-HGPs was increased by 13.5, 17.2, and 282.9%, respectively. In matrices, the heterogeneity of bacteria, fungi, and archaea was decreased by 2.9, 10.9, and 31.1%, respectively. In patches, the heterogeneity of bacteria and archaea was decreased by 6.2 and 19.7%, respectively, whereas the heterogeneity of fungi was increased by 1.4%.

**FIGURE 4:**
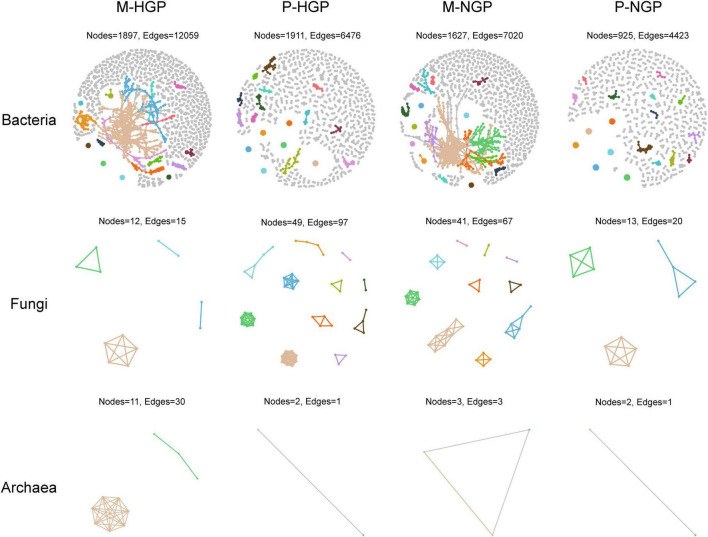
Co-occurrence networks of soil microbial taxa. Visualization of bacterial, fungal, and archaeal connectivity. The top 18 clusters are distinguished by different colors, with other species represented in gray. Nodes indicate individual microbial species and edges represent significant positive Spearman correlations (ρ > 0.9; *p* < 0.01).

Overall, strong correlations of 2,800 operational taxonomic units were observed (Supplementary Figure 2A). In addition, the numbers and correlations of species decreased after GE (Supplementary Figure 2B). For bacteria, the change in cluster number was consistent with the overall changes, which decreased by 1.5 and 14.0% in the matrix and patch, respectively. In addition, the ratio of edges to points decreased from 6.4 to 4.3 in the matrix and increased from 3.4 to 4.8 in the patch, suggesting a decrease in the number of bacterial species, although intermediate correlations increased after GE. For fungi, the number of species clusters increased from 4 to 10 in the matrix but decreased from 11 to 3 in the patch, suggesting that the correlations were inconsistent for changes between fungi and bacteria. The response of archaea to GE was similar to that of bacteria to GE (Figure 4).

### Heterogeneous microbial function

**FIGURE 5:**
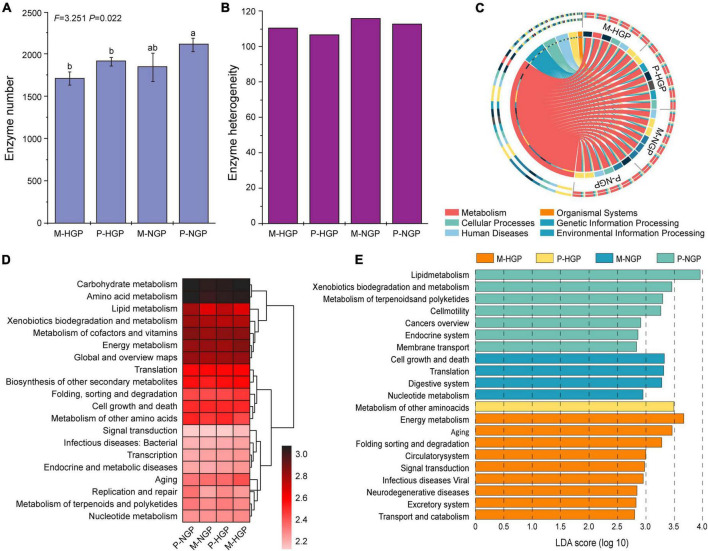
Response of soil microbial function to grazing exclusion. **(A)** The number of enzymes in the four sampling sites (M-HGP, P-HGP, M-NGP, and P-NGP). Different lowercase letters indicate significant differences (*p* < 0.05). **(B)** Heterogeneity of enzymes. **(C)** Proportions of functional microbes (KEGG level 1) in each sample (*n* = 24). **(D)** The relative abundance of the top 20 functions (KEGG level 2), where color depth indicates the relative abundance of genes. **(E)** Functional biomarkers (KEGG level 3) with an LDA score (log 10) of >2.5. Different colors indicate different sampling sites.

Changes in the microbial functional diversity were further analyzed from the perspective of enzymes. As shown in the histogram presented in Figure 5A, the quantity of enzymes was greater in the patch than in the matrix (*P* < 0.05) and this quantity increased after GE, although this increase was not significant, with a *p*-value of 0.07. As shown in Figure 5B, the heterogeneity of enzymes increased by GE, especially in the patch, which was consistent with the changes in heterogeneity of the composition of soil fungi (Figure 3E).

The KEGG database was used for functional depth analysis and to cluster genes with similar functions. Comparisons of our data with the KEGG pathway database (Martínez et al., 2015) suggested that metabolism was the main function of soil microbes, accounting for 71.4% in all genes, followed by cellular processes, environmental information processing, genetic information processing, human diseases, and organismal systems (Figure 5C and Supplementary Figure 3). Furthermore, relatively abundant genes were found to be associated with carbohydrate and amino acid metabolisms (Figure 5D). The LEfSe (Segata et al., 2011) results of the four sampling sites available for functional change analysis identified 9, 1, 4, and 7 functional biomarkers for M-HGP, P-HGP, M-NGP, and P-NGP, respectively. In the HGPs, the relative abundance of genes of other amino acids was greater in the patch than in the matrix. After GE, the relative abundance of genes associated with the KEGG pathways—cell growth and death, translation, digestive system, and nucleotide metabolism—were increased in the matrix, whereas the relative abundance of those associated with the KEGG pathways—lipid metabolism, xenobiotics biodegradation and metabolism, and metabolism of terpenoids and polyketides—were increased in the patch, particularly lipid metabolism, as indicated by an LDA score of 3.95 (Figure 5E). Lipid metabolism pathways included the synthesis and degradation of fatty acids (Supplementary Figure 4). The ability to synthesize and degrade hexadecanoic acid, hexadecenoic acid, octadecanoic acid, octadecenoic acid, and ketone bodies improved.

### Links with localized plant and environmental factors

**FIGURE 6:**
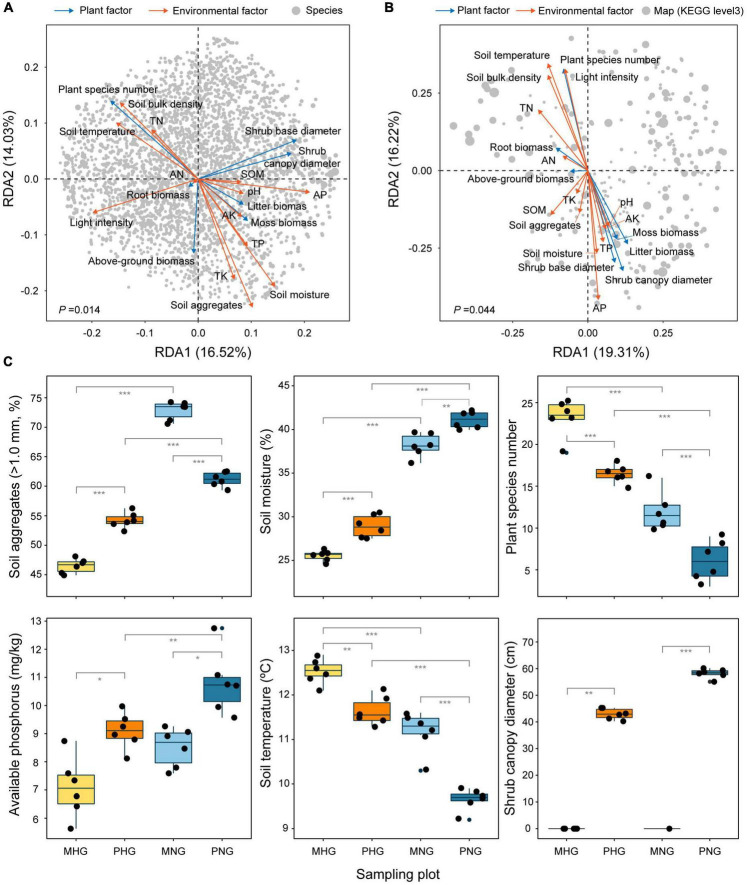
Plant and environmental factors predicted to affect microbial composition and function. Extent of interpretation of **(A)** species distribution and **(B)** function performance by influencing factors. Gray dots indicate microbial species in **(A)** and maps (KEGG level 3) in **(B)**. The dot size indicates the relative abundance of species/functional genes. The cyan arrows indicate the plant factors and the orange arrows indicate the environmental factors in **(A,B)**. AK, available potassium; AN available nitrogen; AP, available phosphorus; SOM, soil organic matter; TK, total potassium; TN, total nitrogen; TP, total phosphorus. **(C)** According to the results in **(A,B)**, relatively important factors were analyzed, including soil aggregates, soil moisture, species number, AP, soil temperature, and shrub canopy diameter. The number of asterisks indicates the level of significance: *p < 0.05, **p < 0.01, ***p < 0.001. The effects of 14 other indicators on GE are shown in Supplementary Figure 7.

RDA is mainly used to show the relationship between taxa or function and environmental factors based on correspondence analysis. Therefore, 20 indices were assessed, which included 7 plot factors (diameter of shrub canopy and base, plant species number, above-ground biomass, root biomass, moss biomass, and litter biomass) and 13 environmental factors (light intensity, temperature, moisture, aggregates, bulk density, organic matter, pH, available nitrogen, available phosphorus, available potassium, total nitrogen, total phosphorus, and total potassium), to identify important drivers (Figure 6A). RDA revealed that soil aggregates, moisture, and plant species number were the most influential factors on microbial composition (Figure 6A), whereas soil available phosphorus, soil temperature, and shrub canopy diameter were the most influential factors on microbial function (Figure 6B). These findings indicated inconsistencies among factors that affect species composition and functional tradeoffs of microbes. In addition, GE was associated with increases in the proportion of aggregates (>1.0 mm), moisture, and available phosphorus and decreases in soil temperature and plant species number (Figure 6C). Light intensity, temperature, moisture, bulk density, available nitrogen, total nitrogen, plant species number, and root biomass showed a positive correlation with each other and a nearly negative correlation with the other 12 indices (Supplementary Figure 5). Among the top 20 family-level taxa, Opitutaceae, Phyllobacteriaceae, and Hypomonadaceae exhibited opposite responses to plant and environmental factors compared with the other 17 indices (Supplementary Figure 6A). Among the top 20 functional taxa (KEGG level 3), ribosome and longevity regulating pathway-multiple species exhibited opposite responses to plant and environmental factors compared with the other 18 indices (Supplementary Figure 6B). These results revealed a difference in relative weights and complex interactions (synergistic and antagonistic changes) among the indices.

## Discussion

Regarding microbial composition, the abovementioned findings support the fertile island theory on HGPs, which suggests that soil resources are more abundant beneath canopies (patches) than outside canopies (matrices) (Bachar et al., 2012). However, GE introduced notable changes in the abundances of bacteria, archaea, and fungi. In contrast, the results of microbial heterogeneity do not support the fertile island theory, which may be associated with grazing intensity, livestock category, and habitats, considering that many soil microbes originate from animal excretions (Liu et al., 2019b; Fang et al., 2021). In addition, shrubs provide hedges for animals, causing livestock to aggregate and walk in the matrix, thereby increasing microbial inputs outside of canopies. In more harsh habitats, shrubs provide shade for animals (Zhang et al., 2019). Moreover, the impact of large farm animals (cattle, sheep, camels, and horses) on shrubs and soil microorganisms differs from that of small animals (wild chickens, lizards, ants, and beetles) (Liu et al., 2015; Stanton et al., 2018). However, soil bacteria, fungi, and archaea are important for plants and environmental feedback. The less persistent effects of plants on soil bacteria compared with fungi (Semchenko et al., 2018; Heinen et al., 2020) may explain why most correlative studies have reported that fungal communities drive changes in plant communities, whereas soil bacteria appear to contribute little to plant–soil feedback. Our results support this finding, as there were no significant differences in bacterial diversity among sampling plots, but changes in fungal diversity were regulated by the environment, and the heterogeneity of fungi was significantly greater than that of bacteria.

Changes in microbial function cannot be equated with changes in community composition. Metabolic plasticity and functional redundancy can explain the relationship between soil microbial composition and function (Louca et al., 2018). The former refers to the ability of microbial taxa to adjust metabolic performance in response to environmental factors without changing composition, whereas the latter indicates that different microorganisms could have similar functions, and alterations in community composition do not necessarily alter functions (Allison and Martiny, 2008; Comte et al., 2013). Although GE reduced microbial heterogeneity and connectivity, microbial community turnover did not alter the functional repertoire of enzymes; thus, functional redundancy was high in the shrub-meadow system. However, changes in microbial community function were more similar to species heterogeneity than to species diversity. The species diversity in P-NGPs was lower than that in M-NGPs, whereas the compositional heterogeneity in P-NGPs was higher than that in M-NGPs. Changes in heterogeneity are consistent with differences in enzyme quantity and heterogeneity in sampling plots. Additionally, some microbial alterations caused by GE were noteworthy, such as the increased relative abundance of Bradyrhizobiaceae, which enhances plant access to atmospheric N (Chen et al., 2016), particularly in matrices, which was consistent with the statistical results of vegetation and the high coverage of *Astragalus przewalskii* (Leguminosae) and *Oxytropis ochrocephala* (Leguminosae) in the M-NGP.

Further analysis of the differences in microbial community functions revealed that fatty acid metabolic capability improved in GE plots. Many fatty acids, such as hexadecanoic acid, hexadecenoic acid, octadecanoic acid, and octadecenoic acid, are allelopathic agents (Iqbal et al., 2019; Kong et al., 2019), which are derived from decomposed litter, root exudates, and stemflows and negatively affect shrubs and the seedlings of other plants (Singh et al., 2021). GE contributed to vegetation recovery by increasing the metabolic capacity of microbes for allelochemicals, which may partly explain the invasion and expansion of shrubs after the removal of grazing pressure (Zhang et al., 2019). However, further evidence is required to verify these findings. For instance, shrub canopies and litters showed a significant and positive correlation with steroid hormone biosynthesis, synthesis and degradation of ketone bodies, and terpenoid backbone biosynthesis. The relative abundance of Pseudomonadaceae was increased by 45.4% in plots after GE. Most Pseudomonadaceae can survive at low temperatures and decompose fatty acids (Chen et al., 2016; Tirloni et al., 2021). Archaea are widely distributed in harsh and extreme environments. Of the 14 species detected in the experimental sites, 8 belonged to the family Methanobacteriaceae, which produce methane (McKay et al., 2019). Compared to bacteria and fungi, lower network connectivity and a lower proportion of shared species were found in archaea. GE decreased the species number and abundance of Methanobacteriaceae, which means GE can potentially improve the methane sink function of soil (Taumer et al., 2021). But soil methane budgets need more information for validation, such as static box experiments and thermogenic methane (Tang et al., 2019; Froitzheim et al., 2021). It is worth noting that archaeal composition was more heterogeneous and more sensitive to GE than bacterial and fungal composition.

Species antagonism and differences in environmental response are important contributors to community heterogeneity. In this study, bacterial diversity was not significantly affected by GE but was rather driven by a decrease in mutualistic microbes and an increase in antagonistic microbes (Gravuer et al., 2020). Opitutaceae, Phyllobacteriaceae, and Hypomonadaceae possibly exist mutualistically but are antagonistic to 13 microorganisms in the top 20 family-level taxa. Fungi are more strongly affected by groundwater than bacteria (Liu et al., 2019a), and the presence of soil phosphorus generally limits most microbial processes (Camenzind et al., 2018). Thus, soil moisture and phosphorus content are important factors that affect microbial composition and function. In the alpine meadow of the Qinghai–Tibet Plateau, bacteria are the dominant members of the soil microbial community. The interactions between plant biomass and diversity and species have important consequences for compositional changes in soil microbial communities (Shi et al., 2020; Yin et al., 2021). Degraded shrub patches retain substantial microbial remnants that drive the subsequent community assembly. Inconsistent feedback of bacteria and fungi in response to GE increases the spatial heterogeneity of microbial communities.

The long-term effects of the degradation or death of shrubs promote the growth of herbaceous vegetation in shrub patches. Degraded or dead shrubs produce a unique ecosystem (Stavi et al., 2021). After GE, canopy sizes were directly proportional to the decreases in light intensity and soil temperature and inversely proportional to the decreases in litter and soil moisture. Patches are more conducive to early plant colonization and seed germination, which has a conservation effect because plants and animals use them as shelters, and in turn, excreta from small animals increase soil fertility (Liu et al., 2019b; Fang et al., 2021). Such “legacy” effects support natural regeneration in severely disrupted areas by improving litter accumulation, maintaining soil moisture, reducing light intensity, and preventing high temperatures (Holle and Tsuyuzaki, 2018). Moreover, the rate of soil erosion was relatively reduced in patches, which is beneficial for vegetation renewal (Lu et al., 2019). However, an excessively large canopy or an excessive period of fencing will diminish conservation efforts and decrease microbial diversity and heterogeneity, which explains why the heterogeneity of soil microbes is relatively reduced in forests compared with grasslands (Eldridge et al., 2020). Plant community composition affects soil microorganisms through root exudates and litter (Gillespie et al., 2021), thereby driving changes in composition and function of soil microorganisms. Therefore, considering the impact of fencing time on the shift in dominant species in plant communities is vital.

The legacy of soil microbes affects vegetation reestablishment (Hannula et al., 2019), with coupled systems exhibiting greater stability than pure forests and grasslands (Rietkerk et al., 2021). Degraded patches have an early promoting effect on the recovery of vegetation after GE, accompanied by the rapid expansion of shrubs. The transition of shrub-meadow systems to shrubland systems results in an overall decrease in soil microbial heterogeneity and a substantial decrease in plant diversity, whereas prolonged GE can induce functional loss of the ecosystem, regardless of functional redundancy. The functional stability of an ecosystem is controlled by the stability of communities and populations that contribute to ecosystem functions (Bluthgen et al., 2016). Inter-species correlations (reciprocity and antagonism) are critical to ecosystem management and prediction of ecological consequences (Yuan et al., 2021). Changes in the abundance of specific functional taxa perhaps reflect ecosystem functioning more accurately than community composition (Kivlin and Hawkes, 2020); thus, the functions of specific taxa and environmental responses should be explored further.

## Conclusion

The results of this study indicate substantial heterogeneity in the composition and function of microbes between patches and matrices, with evident alterations in heterogeneity after GE. Bacterial diversity did not differ significantly among M-HGP, P-HGP, M-NGP, and P-NGP, whereas fungal diversity was significantly higher in P-HGP than in M-HGP. The compositional heterogeneity of fungi was significantly higher than that of bacteria, and GE affected the heterogeneity of microbial composition in the following order: archaea > fungi > bacteria. GE reduced the compositional heterogeneity of bacteria and archaea but showed the opposite effect on soil fungi. GE also increased microbial functional diversity and heterogeneity. In the patch soils, enzyme diversity was greatly improved, and the number of biomarkers increased from 1 to 7 after 6-year fencing. The relative abundances of most genes related to fatty acid metabolism were increased but those of methane-producing archaea were decreased after GE. Soil fungi, which dominated the function of microbial community diversification, accounted for 2.3% of the total microorganisms, but its heterogeneity was significantly larger than that of bacteria. GE, a common practice in grassland management, modulated microbial composition and function by changes in shrub canopy diameter, plant species, soil aggregates, soil moisture, soil available phosphorus, and soil temperature. Our findings revealed high levels of spatial heterogeneity microenvironment in shrub meadows and highlighted the effect of GE on soil microbial composition and function of bacteria, fungi, and archaea.

## Data availability statement

The datasets presented in this study can be found in online repositories. The names of the repository/repositories and accession number(s) can be found below: https://www.ncbi.nlm.nih.gov/, PRJNA795566.

## Author contributions

SW: conceptualization, methodology, data curation, software, funding, and writing—original draft preparation. TA and WW: investigation, reviewing, and editing. XD: methodology, investigation, and reviewing. WL: software, editing, and reviewing. JW: investigation, software, and reviewing. WC: methodology, data curation, software, funding, and editing. All authors contributed to the article and approved the submitted version.

## Footnotes

1
https://cutadapt.readthedocs.io/en/stable/
