# Supplementary material for: Response of soil microbial compositional and functional heterogeneity to grazing exclusion in alpine shrub and meadows in the Qinghai–Tibet Plateau
Source: Front Microbiol. 2022 Nov 30;13:1038805. doi: 10.3389/fmicb.2022.1038805 (PMC9748428; doi:10.3389/fmicb.2022.1038805)
Supplement: Supplementary file 1 [file Data_Sheet_1.PDF]

Supplementary Material

1 Supplementary Figures and Tables

1.1 Supplementary Figures

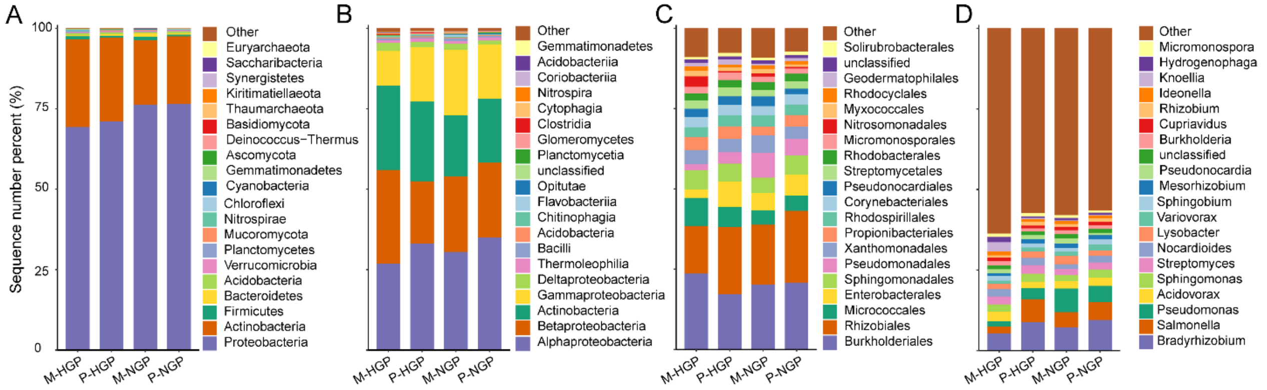

Figure S1. Soil microbial taxa at multiple taxonomic levels. Relative abundance of the top 20 microbial taxa in the soil samples collected from the four sampling sites (M-HGP, P-HGP, M-NGP, and P-NGP) at the phylum, class, order, and genus level (A–D).

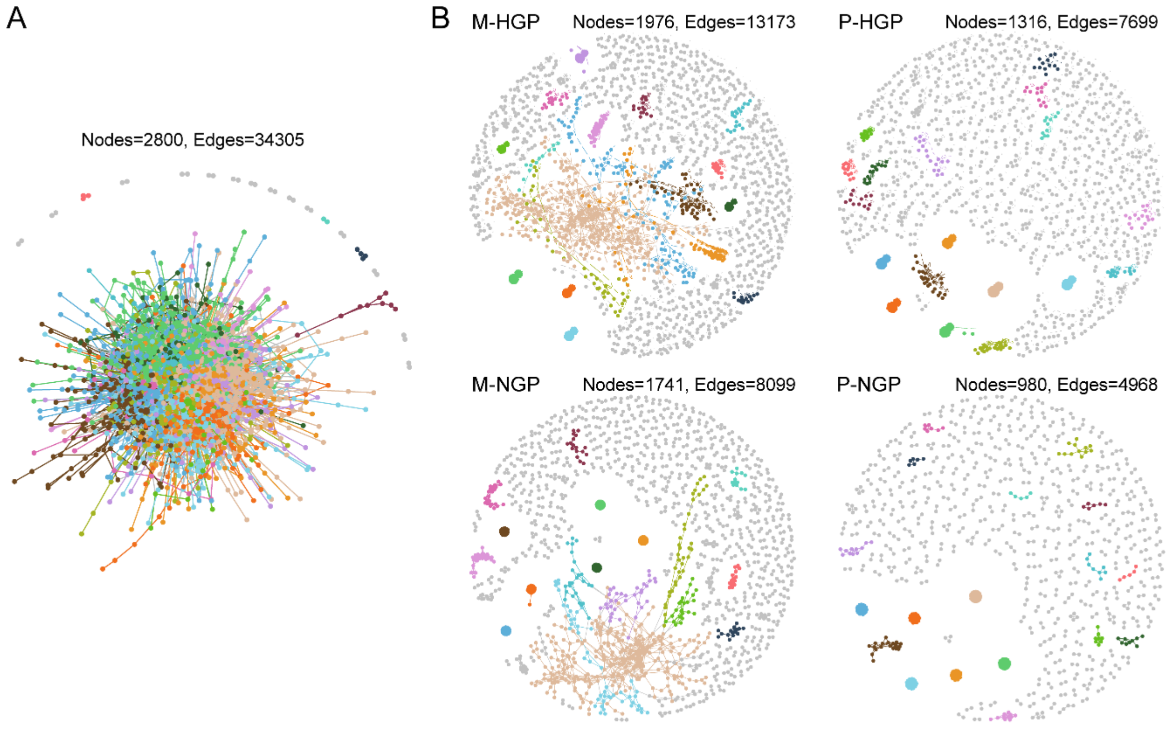

Figure S2. Soil microbial co-occurrence networks. (A) Co-occurrence network including multiple clusters of the relative abundances of OTUs. Dots represent OTUs and lines represent correlations (Spearman, ρ > 0. 9, $p < 0.01$). (B) In M-HGP, P-HGP, M-NGP, and P-NGP, the species co-occurrence network showed observable changes. Dots indicate species and edges indicate correlations (Spearman, ρ > 0. 9, $p < 0.01$). The top 18 clusters are color-coded and other OTUs/species are indicated in gray in (A) and (B).

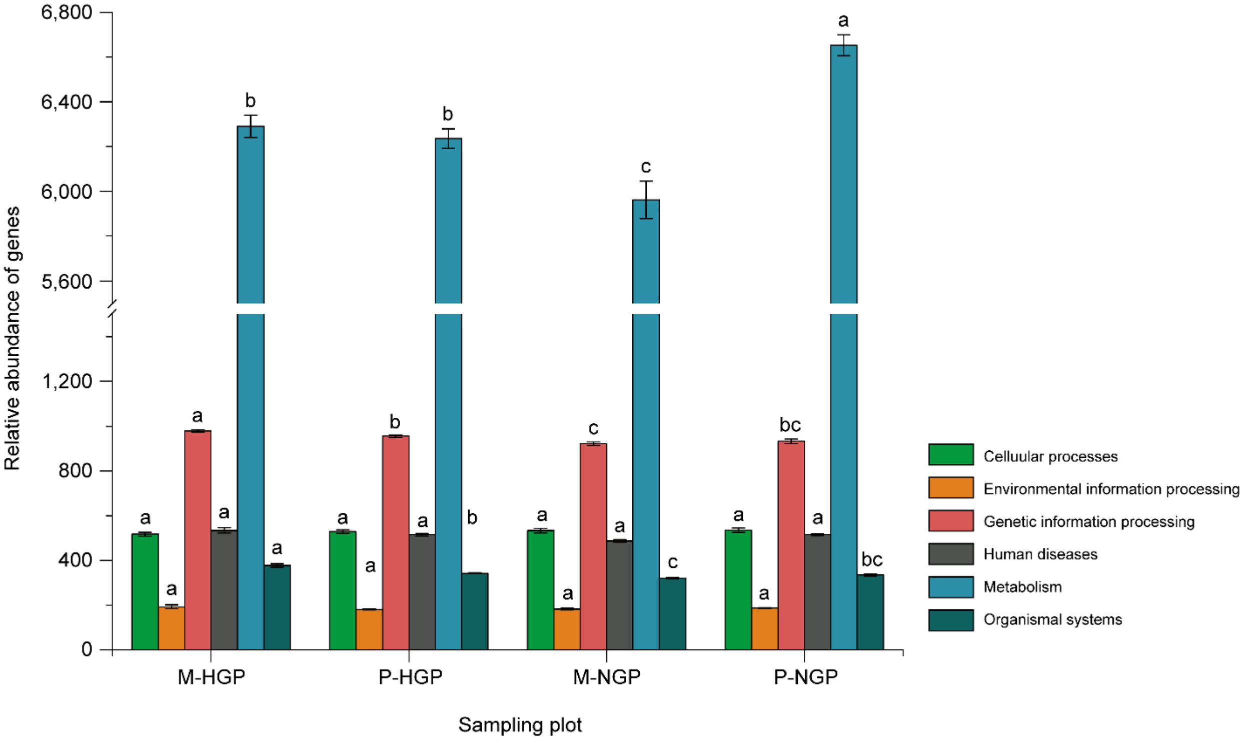

Figure S3. Relative abundances and distributions of genes. Column height indicates relative gene abundance and the length of the line segment indicates the standard error. Different colors indicate six different functions (KEGG level 1). Different lowercase letters indicate significant difference among the four sampling sites ($p < 0.05$).

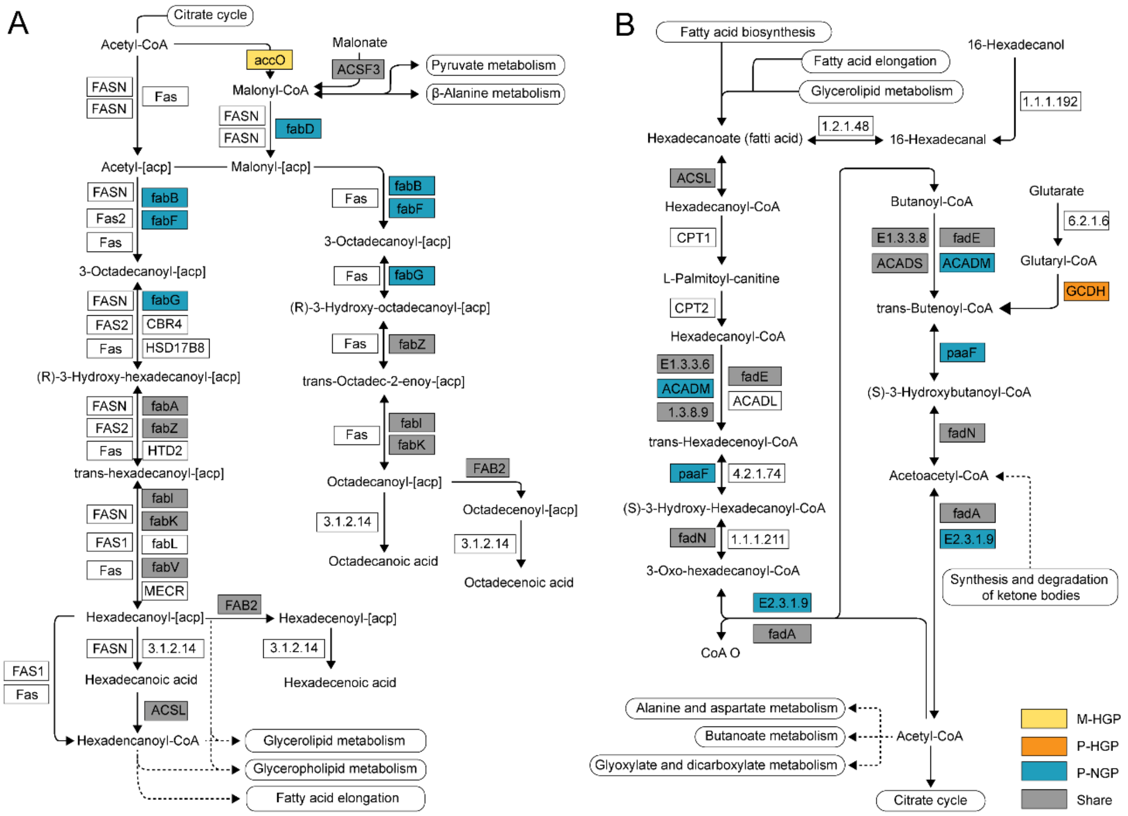

Figure S4. Fatty acid metabolic pathway. (A) Partial KEGG maps associated with fatty acid synthesis and (B) decomposition. Yellow, orange, and cyan indicate unique microbial genes in M-HGP, P-HGP, and P-NGP, respectively. No unique genes were found in M-NGP. Shared genes among the four sampling sites are indicated in gray.

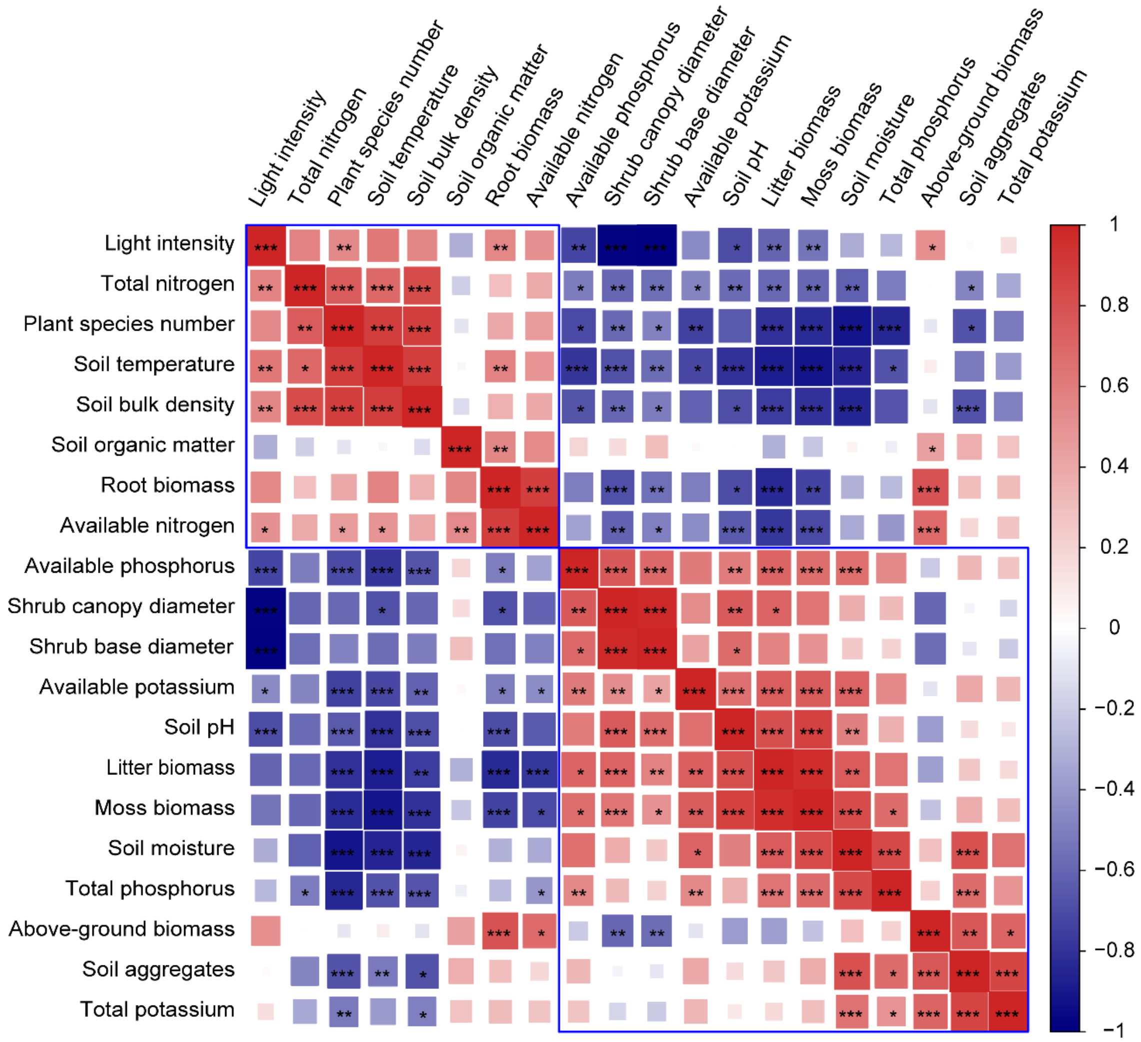

Figure S5. Correlation among influencing factors. The color change from navy to red indicates a change in the Spearman correlation from negative to positive. The color depth represents the correlation coefficient. Blue boxes indicate clusters based on the hierarchical clustering method. The number of asterisks indicates the level of significance: *$p < 0.05$, **$p < 0.01$, ***$p < 0.001$.

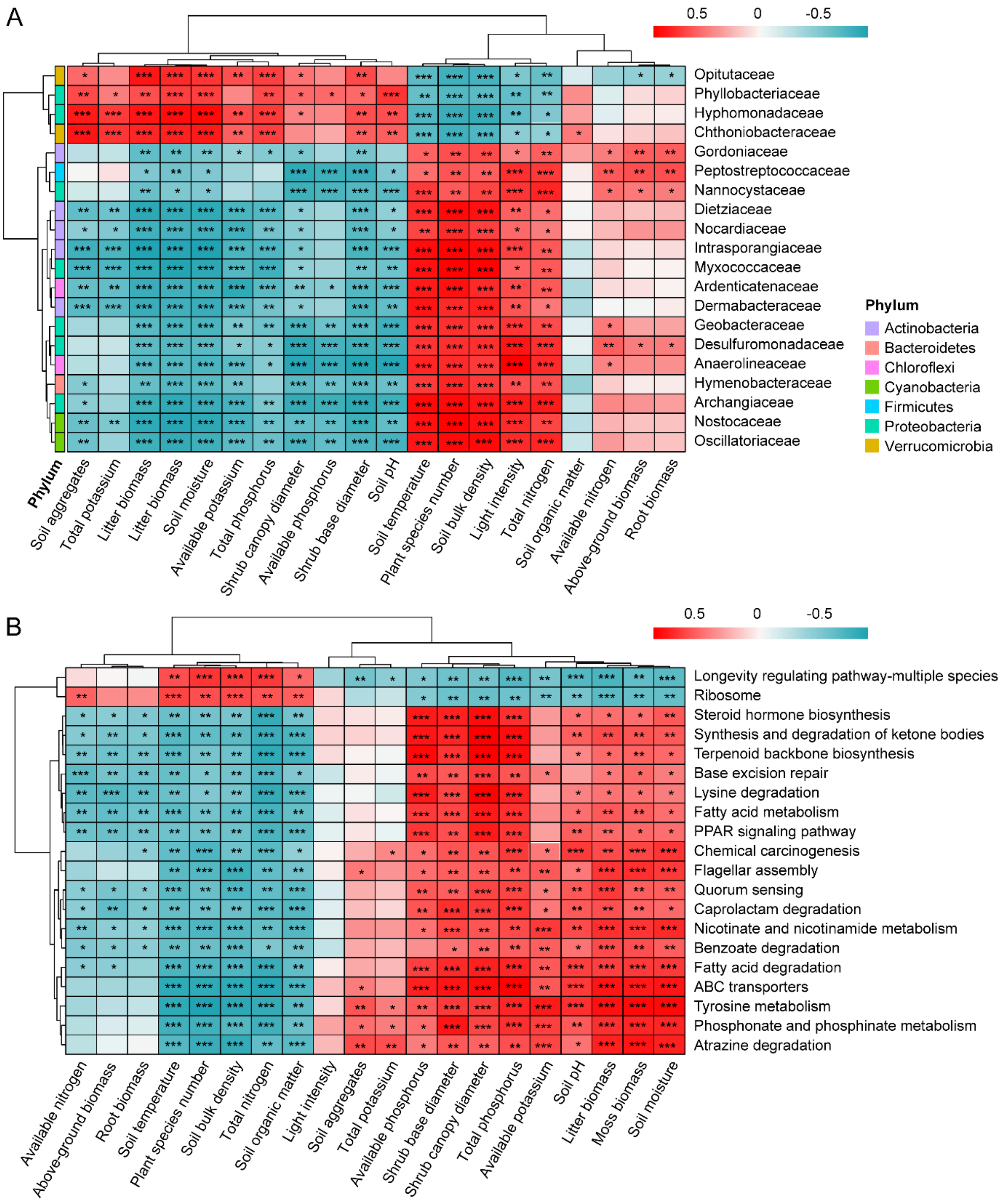

Figure S6. Correlation between microbial composition, function, and factors. (A) Correlations among the top 20 family-level taxa and 20 factors. (B) Correlations among the top 20 functional taxa (KEGG level 3) and 20 factors. Positive effects are indicated in red and negative effects in cyan. The color depth represents the Spearman correlation coefficient (*$p < 0.05$, **$p < 0.01$, ***$p < 0.001$).

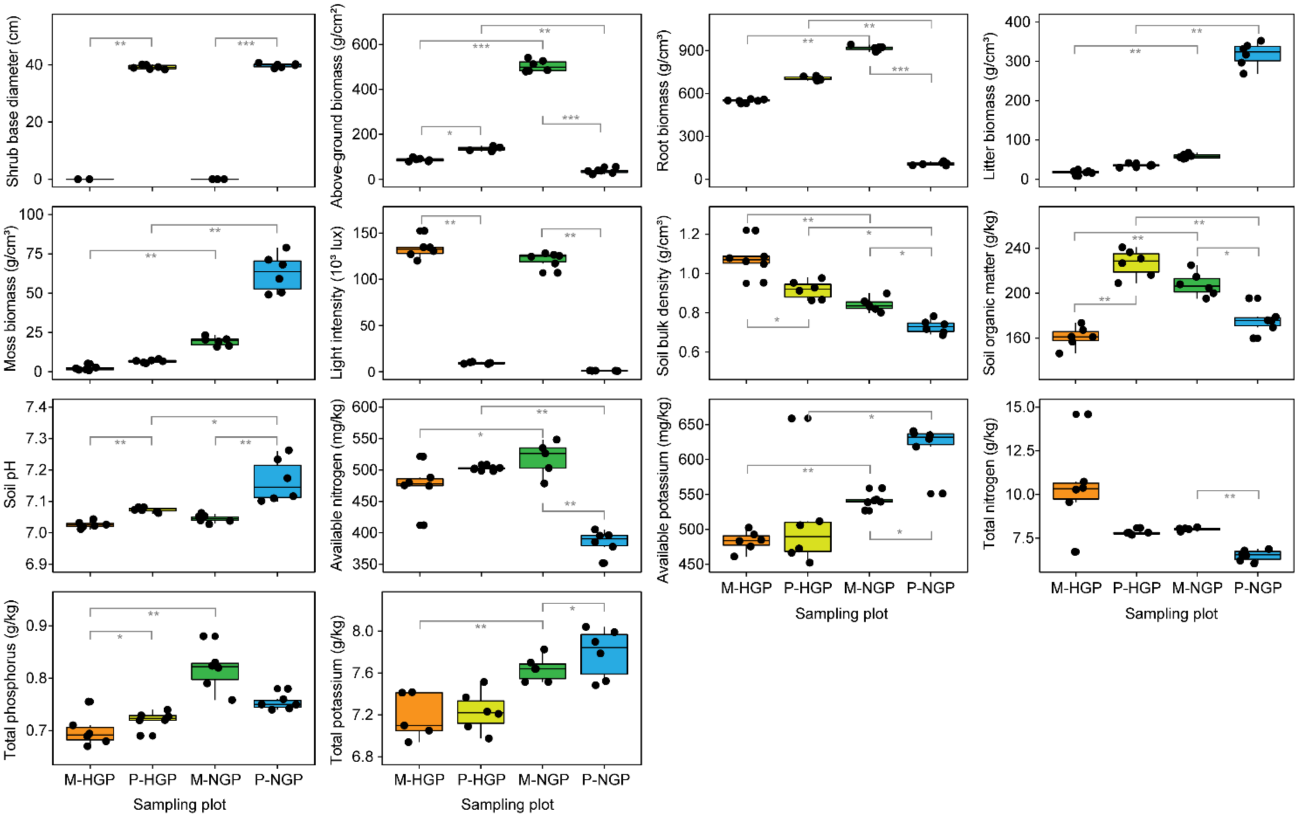

Figure S7. Characteristics of plant and environmental factors (**p* < 0.05, ***p* < 0.01, ****p* < 0.001).

1.2 Supplementary Tables

Table S1 Vegetation characteristics of the experiment site in August 2013.

Items	NGP1	NGP2	NGP3	HGP1	HGP2	HGP3
Shrub coverage (%)	21.8±2.3a	22.5±2.4a	21.1±2.1a	22.8±1.4a	24.3±1.0a	23.8±1.6a
Shrub height (cm)	39.1±4.8a	38.3±3.1a	38.5±5.2a	40.9±6.1a	37.4±6.3a	39.9±3.5a
Grass coverage (%)	79.9±2.1a	81.7±3.6a	79.3±3.7a	80.5±3.3a	77.9±3.0a	81.9±2.4a
Grass height (cm)	6.7±3.7a	6.0±3.6a	7.1±4.3a	5.7±3.9a	5.9±3.8a	6.2±4.1a
Grass biomass (g/m^2)	62.3±9.2a	53.1±14.5a	54.6±5.3a	65.2±9.1a	55.3±6.4a	50.8±8.3a

NGP: non-grazed plot; HGP: Heavily grazed plot. Different lowercase letters indicate significant differences (*P*<0.05).
